# A *Mycobacterium avium* subsp. *paratuberculosis relA* deletion mutant and a 35 kDa major membrane protein elicit development of cytotoxic T lymphocytes with ability to kill intracellular bacteria

**DOI:** 10.1186/s13567-018-0549-3

**Published:** 2018-06-26

**Authors:** Gaber S. Abdellrazeq, Mahmoud M. Elnaggar, John P. Bannantine, Kun T. Park, Cleverson D. Souza, Brian Backer, Victoria Hulubei, Lindsay M. Fry, Samy A. Khaliel, Helmy A. Torky, David A. Schneider, William C. Davis

**Affiliations:** 1 Department of Veterinary Microbiology and Pathology, Washington State University, Pullman, WA USA; 2 Department of Microbiology, Faculty of Veterinary Medicine, Alexandria University, Alexandria, Egypt; 3 USDA, ARS, National Animal Disease Center, Ames, IA USA; 4 Department of Biotechnology, Inje University, Injero 197, Kimhae-si, Gimhae, Gyeongsangnam-do South Korea; 5 Department of Veterinary Clinical Sciences, Washington State University, Pullman, WA USA; 6 Department of Chemistry, College of Arts and Sciences, Washington State University, Pullman, WA USA; 7 USDA, ARS, Animal Disease Research Unit, Pullman, WA USA

## Abstract

Efforts to develop live attenuated vaccines against *Mycobacterium avium* subspecies *paratuberculosis* (*Map*), using indirect methods to screen *Map* deletion mutants for potential efficacy, have not been successful. A reduction in the capacity to survive in macrophages has not predicted the ability of mutants to survive in vivo. Previous studies for screening of three deletion mutants in cattle and goats revealed one mutant, with a deletion in *relA* (Δ*Map*/*relA*), could not establish a persistent infection. Further studies, using antigen presenting cells (APC), blood dendritic cells and monocyte derived DC, pulsed with Δ*Map*/*relA* or a 35 kDa *Map* membrane protein (MMP) revealed a component of the response to Δ*Map*/*relA* was directed towards MMP. As reported herein, we developed a bacterium viability assay and cell culture assays for analysis and evaluation of cytotoxic T cells generated against Δ*Map*/*relA* or MMP. Analysis of the effector activity of responding cells revealed the reason Δ*Map*/*relA* could not establish a persistent infection was that vaccination elicited development of cytotoxic CD8 T cells (CTL) with the capacity to kill intracellular bacteria. We demonstrated the same CTL response could be elicited with two rounds of antigenic stimulation of APC pulsed with Δ*Map*/*relA* or MMP ex vivo. Cytotoxicity was mediated through the perforin granzyme B pathway. Finally, cognate recognition of peptides presented in context of MHC I and II molecules to CD4 and CD8 T cells is required for development of CTL.

## Introduction

*Mycobacterium avium* subspecies *paratuberculosis* (*Map*) is a pathogen with a broad host range. It is the causative agent of Johne’s disease (JD) in cattle, a major disease problem in the US and other countries [1]. Cumulative studies now show humans are susceptible to infection with *Map*. Studies have revealed some infected subjects develop clinical disease manifestations varying from an ileitis in humans referred to as Crohn’s disease, to other diseases associated with autoimmunity [2–5]. To reduce exposure of the public to *Map*, there is a need to find ways to clear dairy herds of *Map*. This has proven difficult, however, because efforts to date have not yielded a vaccine that elicits a protective immune response. This is attributable in part to the lack of methods to accurately predict the efficacy of candidate vaccines before testing in the field.

As illustrated by results from a standardized multi-institutional vaccine study, the use of macrophages and mice as indirect approaches to screen a set of candidate *Map* mutant vaccines for efficacy were not successful [6, 7]. The conclusion drawn from the study suggested more direct methods are needed to fully evaluate the immune response to candidate vaccines in the natural host [7, 8]. Many of the mutants submitted for evaluation in the study were excluded from a vaccine trial in the natural host because they did not exhibit efficacy in macrophage and mouse model systems [8]. This was the fate of the three mutants our group submitted for evaluation in the study. Studies in cattle and goats, however, had shown deletion of one gene, *relA*, interfered with the capacity of *Map* to establish a persistent infection, indicating the mutant was a good candidate for further evaluation [9]. An immune response to the mutant cleared infection and limited the capacity of wild type *Map* to establish an infection [10].

In light of the problems of using indirect methods of assessing the potential efficacy of candidate vaccines, we focused on development of methods to examine the immune response to candidate vaccines in the natural host. We developed an ex vivo platform to study the functional activity of T lymphocytes proliferating in response to live-attenuated and peptide-based candidate vaccines. The first studies conducted with steers vaccinated with Δ*Map*/*relA* demonstrated a CD4 and CD8 T cell recall response could be elicited ex vivo from peripheral blood mononuclear cells (PBMC) stimulated with Δ*Map*/*relA* [9, 11]. Development of a monoclonal antibody (mAb) to CD209, uniquely expressed on blood APC, dendritic cells (bDC), monocyte derived dendritic cells (MoDC), and monocyte derived macrophages (MoMΦ), allowed us to extend the studies and characterize the response in greater detail using APC pulsed with Ag for Ag presentation to T cells [12]. Analysis revealed the recall response could be elicited by antigenic peptides presented by APC pulsed with Δ*Map*/*relA*. Presentation of antigens by APC was blocked by mAbs specific for MHC class I and II molecules showing the recall response was MHC restricted [12].

Further studies were conducted to dissect the recall response and determine which antigens were presented by the APC. This approach revealed a major component of the response was directed towards a 35 kDa membrane protein of *Map*, termed MMP [13]. The recall response to MMP was equivalent to the response elicited by Δ*Map*/*relA* [12]. As reported herein, further analysis of the immune response to Δ*Map*/*relA* and MMP required development of two assays: (1) a bacterium viability assay that was faster than the colony forming unit (CFU) assay for assessment of CTL activity against *Map* and (2) a method to characterize the functional activity of CD4 and CD8 T cells ex vivo. These newly developed assays demonstrated that vaccination with Δ*Map*/*relA* elicits the development of CTL with the ability to kill intracellular bacteria. Further analysis revealed the CTL activity was directed towards MMP. Follow up studies with MMP, ex vivo, demonstrated the same CTL response could be elicited with APC from unvaccinated cattle pulsed with MMP. Analysis the CTL activity revealed cytotoxicity was mediated through the perforin granzyme B (GrzB) pathway.

## Materials and methods

### Animals

Eight Holstein steers were obtained from the *Map* free Washington State University (WSU) dairy herd from 2013 to 2017. In the first phase of the study, two of the steers were vaccinated with the Δ*Map*/*relA* mutant and maintained as a source of blood to characterize cell responses elicited by Δ*Map*/*relA* and MMP. Two additional age-matched naïve steers were maintained as controls. In the second phase of the study, four additional unvaccinated naïve steers were used as a source of blood to conduct the ex vivo studies on the immune response to Δ*Map*/*relA* and MMP. The vaccinated steers were kept in an open feed lot since initial studies demonstrated the mutant Δ*Map*/*relA* was immune eliminated and did not present a health risk to other cattle under study [9]. All the steers were maintained by the college staff. The steers were in good health during the studies. Midway through the initial studies, however, one of the vaccinated steers had to be euthanized because he was unruly and an injury risk to the staff. All protocols were approved by the WSU Institutional Animal Care and Use Committee (ASAFs 3360 and 04883).

### Preparation of *Map* K10, K10_GFP_, Δ*Map*/*relA*, and MMP

The Δ*Map*/*relA* mutant was constructed in the K-10 and K10_GFP_ strains of *Map* using site directed allelic exchange, as previously described [14]. Cultures of *Map* K10, K10_GFP_, and Δ*Map*/*relA* were prepared from single colonies and used to inoculate Middlebrook 7H9 broth flasks (Difco, BD biosciences, USA) supplemented with 6.7% para-JEM GS (Trek Diagnostic Systems, OH, USA), 2 μg/mL mycobactin J (Allied Monitor, MO, USA), and 0.05% Tween 80 (Sigma-Aldrich Corp.) [9, 14]. The cultures were expanded on a shaking stand at 37 °C. When the broth cultures reached an OD_600_ of 0.6–0.8, master stocks were prepared in 1.5 mL micro-centrifuge screw-cap tubes for immediate use in each experiment. To ensure a single-cell suspension, bacterial stocks were disaggregated by passages through a 26-gauge needle three times, then the bacterial numbers were estimated based on the final OD_600_ values [14].

The full length MMP is encoded by MAP2121c in the K-10 genome [15]. It was expressed in ClearColi as a maltose-binding protein for purification [16].

### Bacterium viability assay

The *Map* viability assay was adapted from a method developed by Kralik et al. [17] to distinguish and quantify the concentration of live *Map* present in food products and samples from the environment. The method involves the use of propidium monoazide (PMA), a membrane impermeant fluorescent compound similar to propidium iodide. It only enters dead cells and intercalates into DNA [18]. When it is activated by light, it binds covalently to DNA and blocks binding of probes used to detect *Map* genes. The concentration of live *Map* in a mixture of live and dead bacteria is determined from a standard curve generated with known concentrations of pure DNA from live *Map*. The number of live bacteria present in a sample preparation is determined by using quantitative PCR (qRT-PCR) with a probe specific for a single copy gene, F57, specific for *Map* [17]. Two types of controls were used to adapt the PMA method for measuring *Map* killing. The first set of controls was used to demonstrate the concentration of live *Map* in a defined mixture of live and dead bacteria, which was determined from a standard curve generated with DNA from a known number of live *Map*. The controls were prepared from pure DNA from live *Map* for use as reference standards for extrapolating the extent of killing mediated by Ag-specific CTL. In the first set of controls, tubes were prepared to contain 2 × 10^7^ *Map*/ tube. One set was heat-killed by incubating *Map* at 90 °C for 15 min. Tubes were then prepared to contain 100% live, 50% live/50% killed and 100% killed *Map* suspended in 400 μL of H_2_O in 1.5 mL translucent Eppendorf tubes and stored at −20 °C until processed. The second set of controls was used to quantitate the number of live *Map* present in *Map*-infected MoMΦ (generation of MoMΦ described below). This set of controls was essential for determining the extent of killing by CTLs since it covered the dynamic range for detection of live vs dead *Map* obtained from infected MoMΦ before and after incubation with CTL. Aliquots of *Map* mixed in four ratios, 100% live, 75% live/25% killed, 25% live/75% killed, and 100% killed, were prepared and added to the cultures of MoMΦ at a MOI of 10 respectively and incubated for 3 h. The cultures were then washed to remove free bacteria. In this set of controls, cells were lysed following infection and incubation with *Map* by adding 2 mL of 0.01% saponin in H_2_O and incubating at 37 °C for 15 min. The cell lysates were centrifuged for 30 min at 4500 rpm to pellet the bacteria. The supernatants were discarded and the pellets re-suspended in 1 mL H_2_O and transferred into micro-centrifuge tubes. The second centrifugation step was performed to harvest the washed bacteria at 14 000 rpm for 10 min. The supernatants were discarded, and the pellets re-suspended in 400 μL of H_2_O in 1.5 mL translucent Eppendorf tubes and stored at −20 °C until used.

### Treatment with PMA to block binding of the F57 probe

The basic procedure for PMA treatment was carried out according the manufacturer’s instructions. 1 µL of 20 mM PMA working stock solution was added to 400 µL of the previously prepared preparations of cell controls to reach a final dye concentration of 50 µM. The translucent PMA-treated tubes were incubated at room temperature for 5 min in the dark on a rocker followed by brief spinning. To avoid overheating during exposure to the halogen light, a plastic tray was prepared with a frozen ice pack wrapped in aluminum foil. The tubes were placed in a diagonal slant position on top of the ice pack. The tray was then set on a rocking platform to ensure continuous mixing during light exposure. Light exposure was performed for 5 min using a halogen lamp with a 650 W bulb placed at a distance of ~20 cm from the tubes. Cells were subsequently harvested by centrifugation at 10 000 ×* g* for 5 min. The supernatants were discarded and the cell pellets from the two sets of controls processed for isolation of DNA [19].

### DNA extraction

DNA was extracted using the protocol for Gram-positive bacteria using DNeasy^®^ Blood and Tissue kit (Qiagen, USA) following enzymatic lysis to facilitate breakdown of the *Map* cell wall as described by Park et al. [19]. In all cases, DNA extraction was performed in duplicate. The DNA was eluted in 100 μL of the designated elution buffer in the kits. DNA yield was measured by a NanoDrop^®^ ND-1000 Spectrophotometer (Thermo Fisher Scientific Inc., Waltham, USA) using A_260/280_ ratio with 1 μL of sample.

### Quantitative real-time PCR

We used the TaqMan RT-PCR method, targeting the single copy F57 gene specific for *Map* (F57 qPCR) to quantify the relative concentration of live *Map* in all samples [20]. The StepOnePlus Real-Time PCR System machine (Applied Biosystems, CA, USA) was used to collect the data. Each DNA sample was diluted to 1 ng/μL and run in duplicate. The total reaction volume was 25 μL including 5 μL of the DNA sample. The qPCR conditions and sequences for primer and probe were the same as previously described [19]. The reactions were run for 40 cycles. Genomic DNA (gDNA) prepared from a pure culture of *Map*, was used to generate the standard curve using the F57 probe. Eight dilutions of DNA were used, starting with 4 × 10^7^ copies diluted down to 4 copies. The mass of one copy of *Map* gDNA was calculated using the following equation:

$$m = \left( n \right) \times \left( {1.096 \times 10^{ - 21} \;{{\text{g}} \mathord{\left/ {\vphantom {{\text{g}} {\text{bp}}}} \right. \kern-0pt} {\text{bp}}} } \right),$$

where *n* is the genomic size and *m* is the mass of the genome [19]. The qPCR results were analyzed using StepOne Software v2.1 (Applied Biosystems). A reduction in signal correlates with a reduction in the live *Map* present in a preparation of *Map* containing live and dead *Map*. The frequencies of viable *Map* present in the samples were determined based on the means of the threshold (C_T_) values as described by Kralik et al. [17]. C_T_ values are inverse to the amount of amplified target gene in the tested sample (lower C_T_ values indicate a higher concentration of the targeted gene and vice-versa).

### Ex vivo assays to characterize the functional activity of CD4 and CD8 T cells

**Figure 1:**
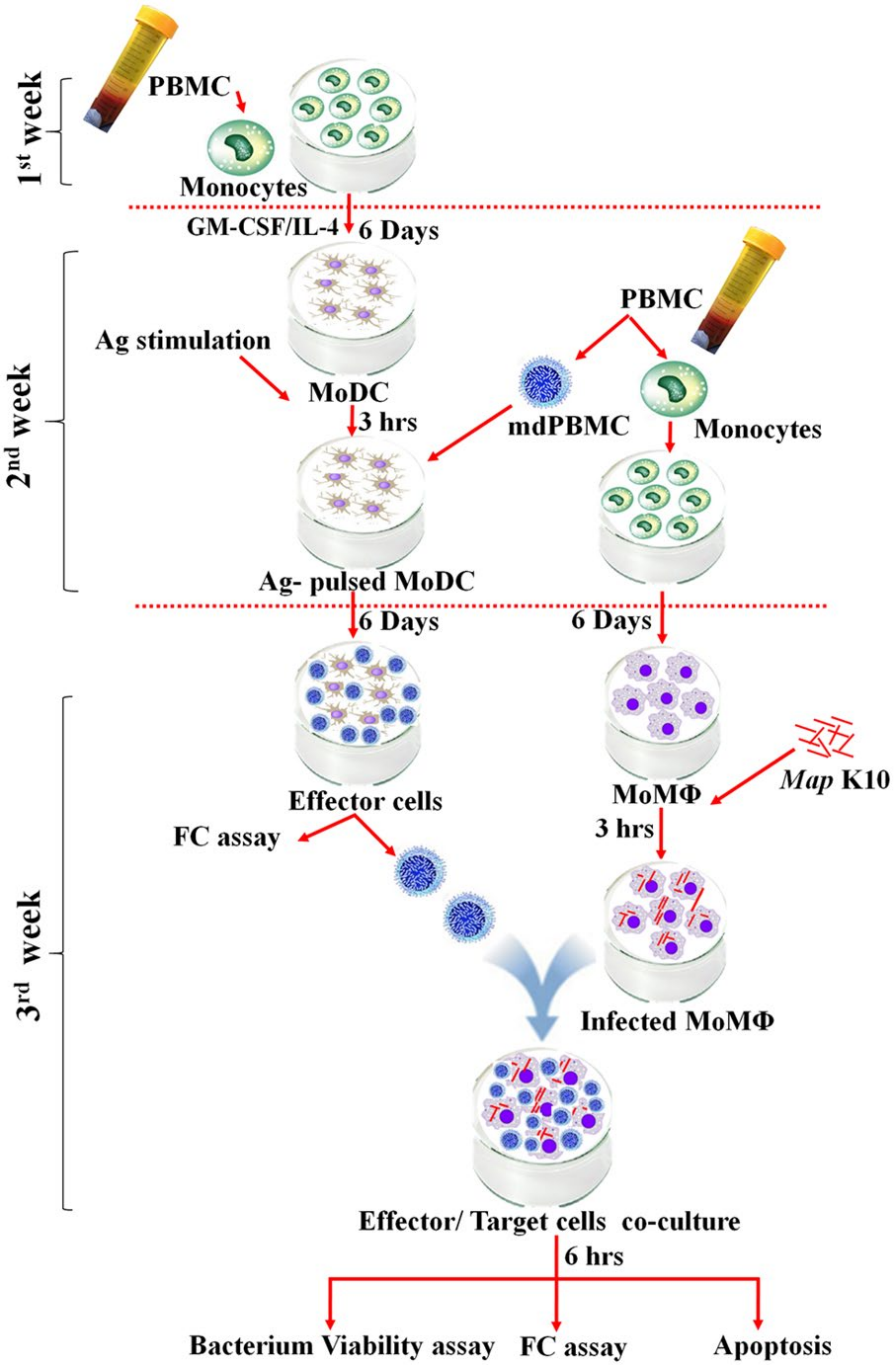
**Protocol for testing activity of CD4, CD8 T cells stimulated with Ag-pulsed bDC, MoDC.** See “Materials and methods” for detail.

The flow diagram in Figure 1 illustrates the ex vivo protocols used to conduct the studies with cells obtained from the vaccinated and unvaccinated steers.

### Generation of MoDC and CTL from PBMC from the vaccinated and unvaccinated control steers

As illustrated in Figure 1, the first blood draw from the vaccinated and unvaccinated control steers was used to generate MoDC for antigen (Ag) presentation to monocyte depleted PBMC (mdPBMC). Magnetic microbeads coated with a cross-reactive anti-human CD14 mAb were used to isolate monocytes per the manufacturer’s instructions (Miltenyi Biotec) [21]. The average purity of isolated CD14^+^ cells was greater than 98%, as determined by FC analysis using an anti-bovine CD14 mAb, CAM36A [11, 22]. Monocytes (2 × 10^6^) were added to wells of six well culture plates and cultured in 3 mL of complete culture medium (cRPMI) [RPMI-1640 medium with GlutaMAX™ (Life Technologies, CA, USA) supplemented with 10% calf bovine serum (CBS), 1 mM β-mercaptoethanol, 100 units/mL of penicillin G, and 100 μg/mL of streptomycin sulfate] in the presence of a DC growth cocktail containing bovine GM-CSF and IL-4 (Kingfisher Biotech, MN, USA). On the 3rd day, 1.4 mL of the medium was replaced with 1.8 mL of fresh medium containing the cocktail. The cultures were incubated for an additional 3 days to obtain MoDC. Δ*Map*/*relA* (5 × 10^6^ *Map*/well) or MMP (5 μg/mL) were added to the cultures of MoDC and incubated for 3 h at 37 °C, 5% CO_2_. The adherent MoDC were then carefully washed 3 times with warm RPMI (without antibiotics) to remove free bacteria or MMP.

Following preparation of fresh PBMC from the second blood draw of the vaccinated and control steers, one portion was used to prepare monocyte depleted PBMC (mdPBMC), as described, using magnetic beads coated with anti-CD14 to remove monocytes. The mdPBMC, were added to their respective autologous MoDC pulsed with Δ*Map*/*relA* or MMP (2 × 10^6^/mL in 5 mL of cRPMI). Following 6 days of culture, the cells were collected and used in FC and the *Map* killing assay. The second portion of fresh PBMC was used to generate MoMΦ as described below for use in the ex vivo studies.

### Generation of MoDC and CTL from PBMC from the unvaccinated steers

Two rounds of antigenic stimulation were used to generate CTL from mdPBMC from the unvaccinated steers. The same general protocol was used as illustrated in Figure 1. The main difference is that the first blood draw from the unvaccinated steers was used for (1) the first round of stimulation of mdPBMC with Δ*Map*/*relA* and MMP, processed and presented by bDC (present in the mdPBMC) and (2) to generate MoDC for use in the second round of antigen (Ag) presentation to mdPBMC by MoDC pulsed with Δ*Map*/*relA* or MMP. The second blood draw was used to generate MoMΦ for use as target cells. The rest of the protocol was identical to the protocol described in Figure 1.

### Generation of MoMΦ for use as target cells

At the times indicated in the protocol, fresh PBMC were re-suspended in cRPMI, placed in 150 mm tissue culture plates and incubated overnight. The majority of the non-adherent cells were then removed the following day. The adherent cells were cultured in fresh medium for 4 days then brought into suspension by incubating them on ice in the presence of EDTA in PBS [4 mL EDTA (250 mM stock in H_2_O), 5 mL CBS, 91 mL PBS]. The cells were re-suspended in cRPMI, counted, and then used to prepare MoMΦ for scanning confocal microscopy or use in the viability assay.

### Infection of MoMΦ with *Map* K10_GFP_ for scanning confocal microscopy

A two chamber culture slide was used to prepare cultures of MoMΦ infected with *Map* K10_GFP_ to determine whether a 3 h incubation time is sufficient for preparing infected MoMΦ for use in the intracellular killing assay. MoMΦ were added to the two chambers (~2 × 10^4^ MoMΦ/well) of the culture slide. After 2 days of additional culture, the MoMΦs were infected with K10_GFP_ at a MOI of 10:1 (2 × 10^5^ K10_GFP_ to ~2 × 10^4^ MoMΦ) in antibiotic free cRPMI. The slide was incubated at 37 °C, 5% CO_2_ for 3 h. Immediately following incubation, the medium containing free bacteria was washed away with warm cRPMI. The cells were then treated with a saponin-based permeabilization and wash reagent (BD Pharmingen) for 30 min at room temperature, then washed once with 1 mL of PBS. The cells were counter stained for 5 min with 500 µL of diluted (1:3000) propidium iodide stain (1 mg/mL stock solution, Invitrogen). Subsequently, the cells were rinsed several times in PBS then fixed with 2% formaldehyde in PBS overnight at 4 °C. After fixation the cells were covered with Vectasheild antifade agent and imaged on a Leica SP8 scanning confocal microscope, using the Leica Application Suite X software 1.10.12420.

### Infection of MoMΦ for use in the viability assay

MoMΦ were distributed into six well culture plates (2 × 10^6^ cells/well) and cultured for an additional 2 days and then used as target cells.

MoMΦ were infected with *Map* at a multiplicity of infection (MOI) of 10:1 (2 × 10^7^ *Map* to ~2 × 10^6^ MoMΦ/well) in antibiotic free cRPMI. The culture plates were centrifuged at 700 ×* g* for 5 min, then incubated at 37 °C, 5% CO_2_ for 3 h. Extracellular bacteria were removed by washing 5 times with warm RPMI with no antibiotics. A vacuum source, equipped with a side-arm flask, rubber tubing and a pipette tip, was used for liquid removal in culture wells to avoid detaching the adherent MoMΦ. Two wells from each of the respective sets of 6 wells, containing *Map* infected MoMΦ, were used as controls, without addition of primed or unprimed preparations of mdPBMC.

### Incubation of mdPBMC from the vaccinated and the control steers with MoMΦ alone or infected with *Map*

mdPBMC prepared from the vaccinated and control steers as outlined in Figure 1, were added to the cultures of MoMΦ alone or MoMΦ infected with *Map* (2 × 10^6^ cells/well). After incubation for 6 h, the non-adherent cells were washed away with warm RPMI. The remaining MoMΦ were collected and processed for the *Map* viability assay.

### Incubation of mdPBMC from unvaccinated steers with MoMΦs, alone or infected with *Map*

mdPBMC preparations from the unvaccinated steers prepared according to the protocol, were added to the preparations of MoMΦ alone or infected with *Map* (2 × 10^6^ cells/well) and incubated for 24 h. The non-adherent cells were collected and held separately until collection of the adherent cells and then recombined for analysis of *Map* viability.

### Cell processing for the *Map* viability assay

Cells in one control well of infected MoMΦ were lysed immediately following infection with *Map* using 0.01% saponin in H_2_O as described. The pellets were re-suspended in 400 μL of H_2_O in 1.5 mL translucent Eppendorf tubes and stored at −20 °C for later analysis. The other control well was incubated for 6 or 24 h concurrent with infected MoMΦ cultures overlaid with Ag-primed or unprimed preparations of mdPBMC. At the end of respective incubation times, all cultures were lysed as described. All lysed cells were treated with PMA and subjected to DNA extraction followed by qPCR as previously described in the viability assay to determine the extent of intracellular killing.

### Intracellular labeling for perforin and GrzB

mdPBMC, prepared according the protocol (Figure 1) and co-cultured with *Map*-infected and non-infected MoMΦ for 24 h, were used to determine the frequency of CD4 and CD8 T cells positive for perforin and GnzB following incubation. In these sets of cells, Brefeldin A (BFA; 1 μg/mL, BD Biosciences) was added during the last 12 h of incubation to block secretion of proteins. The intracellular staining was carried out as described [23] with minor modification. In brief, the non-adherent cells were harvested and labeled for surface staining. Then, cells were fixed and permeabilized using BD Cytofix/Cytoperm fixation permeabilization solution kit (BD Bioscience) for 30 min in the dark on ice followed by washing in Perm/Wash buffer. The cells were re-suspended in Perm/Wash buffer and labeled intracellularly with different combinations of the mAbs shown in Table 1. Data were collected on a modified FACS Calibur DxP8 Analyzer equipped with a FlowJo operating system (Cytek Biosciences Inc. Fremont, CA, USA).

**Table 1:** **MAbs used in the present study**

mAb	Isotype	Specificity/source	Fluorochrome	Labeling
ILA11A	IgG2a	CD4 WSUMAC	PE CY5.5	Indirect
CACT138A	IgG1	CD4 WSUMAC	Alexa Fluor^®^ 647	Indirect
7C2B	IgG2a	CD8 WSUMAC	PE CY5.5	Indirect
CACT80C	IgG1	CD8 WSUMAC	Alexa Fluor^®^ 647	Indirect
ILA116A	IgG3	CD45R0 WSUMAC	Alexa Fluor^®^ 488; PE	Indirect
CAM36A	IgG1	CD14 WSUMAC	Alexa Fluor^®^ 647	Indirect
209MD26A	IgG2a	CD209 WSUMAC	PE CY5.5	Indirect
δG9	IgG2b	Perforin Biolegend	PE	Direct
B-D48	IgG1	Perforin Biolegend	PE/CY7	Direct
351927	IgG2a	Granzyme B R&D Systems	PE	Direct

### Apoptosis

Aliquots of *Map* infected MoMΦ incubated with Ag-primed mdPBMC for 24 h were collected and used to examine the role of apoptosis in intracellular killing of *Map*, using a PE Annexin V Apoptosis kit (BD Pharmingen). [The kit includes Annexin V conjugated with phycoerythrin (PE) and 7AAD, a fluorescent membrane impermeant dye that is taken up by dead cells with a permeant membrane. Annexin V detects the changes in the membrane during the early stages of apoptosis. As apoptosis progresses with disruption in the integrity of the membrane, cells become double positive, necrotic]. Non-adherent cells comprised of lymphocytes and MoMΦ, from each culture were collected and held in suspension. The adherent cells from each cell culture were suspended and added to the corresponding preparation of non-adherent cells. The cells were pelleted by low speed centrifugation and re-suspended in PBS. Approximately 1 × 10^6^ cells were transferred into 5 mL tubes and washed twice with cold PBS. Cells were then labeled according to the manufacturer’s instructions. Two types of controls were included in this assay (1) uninfected MoMΦ alone and (2) uninfected MoMΦ incubated with MMP-primed mdPBMC. The cell preparations were analyzed by FC within 1 h of labeling by gating only on MoMΦ. At least 10^5^ gated events were collected for each cell preparation.

One set of the cultures used for the apoptosis study, as described above, was maintained for 3 days after initiation of co-culture of mdPBMC, to determine whether necrosis or apoptosis increased over time.

### Flow cytometry (FC)

**Figure 2:**
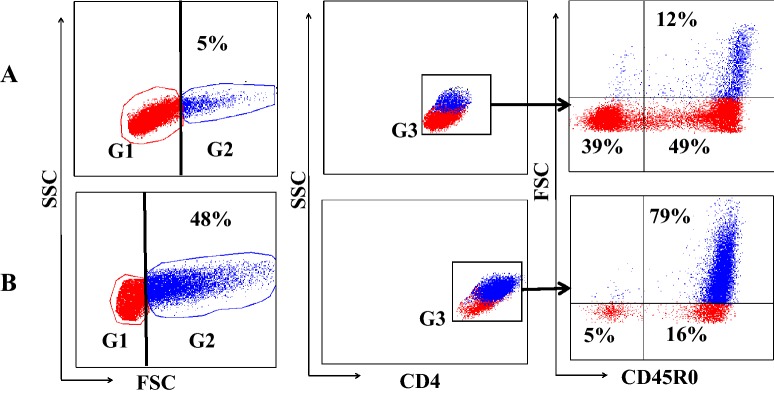
**Flow cytometric gating strategy for analysis CD4, CD8 T cell response to Ag-pulsed bDC/MoDC.** Gates were placed to define small lymphocytes (G1) and large activated lymphocytes (G2) and color coded blue to track activated lymphocytes. A third gate was placed on CD4 cells in this illustration to isolate activated cells for analysis. Visualization in forward scatter (FSC) vs CD45R0 (memory) shows activated cells that increase in size appear in G2. **A** mdPBMC cultured in medium alone for 6 days. **B** mdPBMC following Ag stimulation and culture for 6 days. Comparison of cells in **A** with **B** show only Ag specific CD4 memory T cells proliferated in response to stimulation with MMP. This population also expressed CD25, an indication of cells in this gate are activated (not shown). Only the activated proliferating cells in the upper right quadrant were selected for data analysis. Gated CD8 T cells had equivalent profiles.

Cells, prepared for FC analysis at the times indicated in the protocol (Figure 1), were washed one time in PBS/ACD, centrifuged, then re-suspended in serum-free RPMI and counted. For cell labeling, cells were distributed in 96 well polystyrene V-shape bottom microplates (10^6^ cells/well). Combinations of mAbs (Table 1), obtained from the WSU Monoclonal Antibody Center (WSUMAC), were used for labeling as previously described [24]. Data were collected on a FACSort (BD Immunocytometry systems, San Jose, CA, USA) equipped with Cell Quest software. The gating strategy used to collect the data is shown in Figure 2.

### Statistical analysis

Data were imported into JMP software (version 12.0.1; SAS Institute Inc., Cary, NC, USA) for statistical analysis and graphical presentation (means and standard deviations). Two-way ANOVA was used to conduct statistical analyses and included an interaction term expect for the analysis of data represented in Figure 6B. In this instance, the second factor (blood donor) was instead considered a blocking factor. Post-hoc multiple comparisons were conducted using Tukey HSD (overall α = 0.05).

## Results

### Development of a PMA-based viability assay to quantify the relative concentration of live *Map* in a mixed population of live and dead bacteria

**Figure 3:**
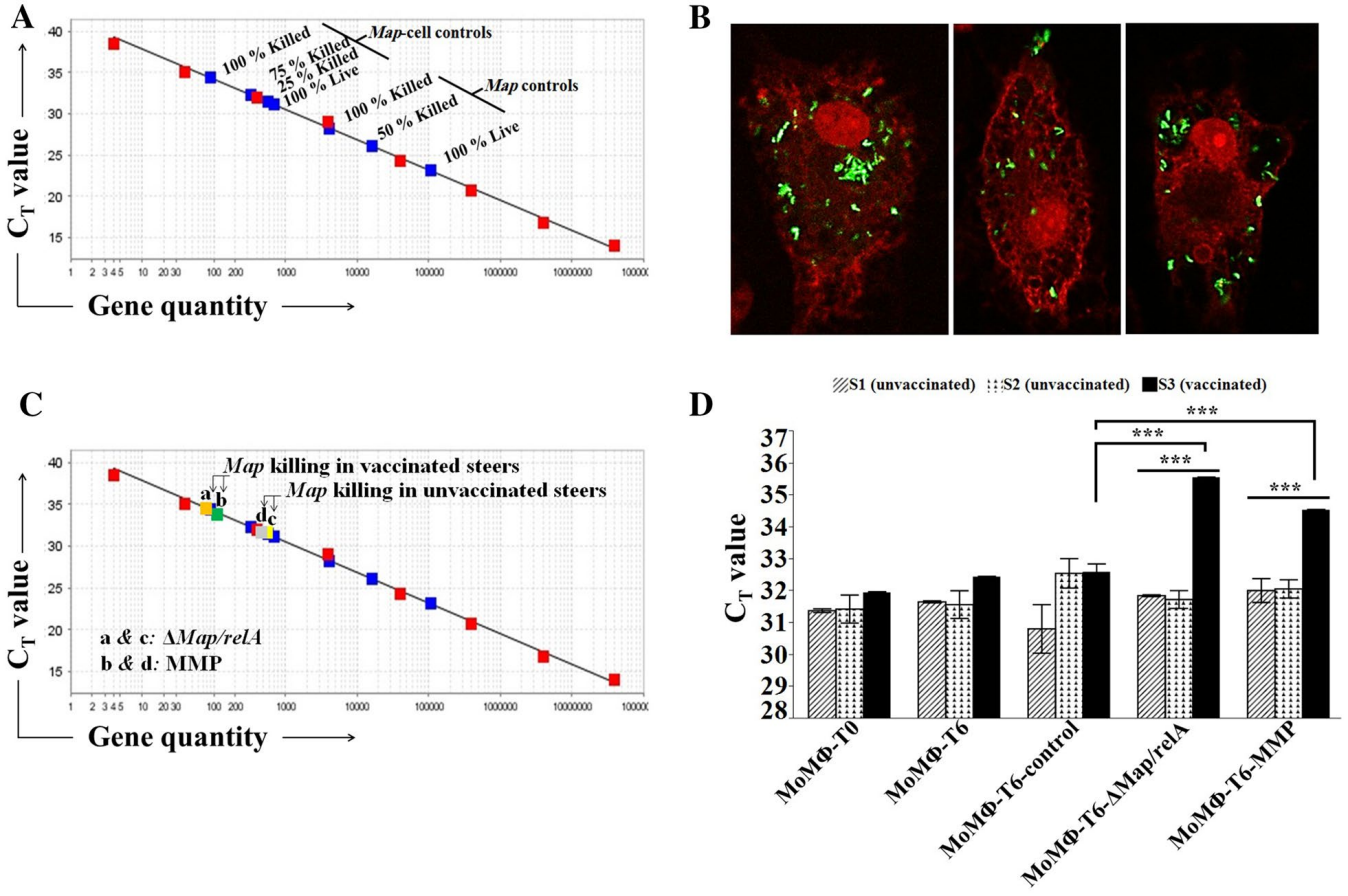
**Killing assay. A** Standard curve with 8 dilutions DNA from live *Map* (red boxes) 4 × 10^7^ to 4 copies. Blue square data points represent DNA from two sets of controls; the lower set of squares are from *Map* DNA (2 × 10^7^ *Map*) prepared from mixtures of 100% live, 50% live/50% dead, and 100% dead bacteria after PMA treatment. Upper four squares are from DNA isolated from *Map* after 3 h incubation with MoMΦ. Squares represent mixtures of *Map* used to infect MoMΦ, prepared at 2 × 10^7^ *Map*: 100% live, 75% live 25% dead, 25% live 75% dead and 100% dead *Map*. C_T_ values represent average of duplicate preparations of DNA. **B** Confocal microscopy showing abundance of K10_GFP_ taken up during 3 h incubation with MoMΦ. **C** Killing by effector cells plotted on standard curve from **A**. C_T_ values of mdPBMC from vaccinated steer with MoDC-pulsed Δ*Map*/*relA* (a: gold box) or MMP (b: green box) and mdPBMC from one control steer stimulated with MoDC pulsed with Δ*Map*/*relA* (c: yellow box) or MMP (d: gray box). **D** Summary of 6 replications of killing assay comparing killing activity mdPBMC from vaccinated and control steers. Mean and standard deviation for each treatment effect (*n* = 6 independent experiments). Two-way ANOVA was significant (*F* = 88.3205; *P* < 0.0001) and included significant interaction effect between treatments and steer-status (*F* = 46.1411; *P* < 0.0001). Within unvaccinated steer S1 and vaccinated steer S3, mean effects of T6-∆*Map*/*relA* and T6-MMP were significantly greater than T6-control (each, *P* < 0.0001; ***S1 not shown). Mean effects within unvaccinated steer S2 either smaller (T6-∆*Map*/*relA*, *P *= 0.0027; *not shown) or not different (T6-MMP; *P* = 0.3668) than T6-control. Effect of T6-∆*Map*/*relA* or T6-MMP for vaccinated steer S3 was significantly greater than effect for either unvaccinated steer (each, *P* < 0.0001).

Evidence obtained in previous studies designed to study the effector T cell response to *Mycobacterium tuberculosis* (*Mtb*), used the CFU method to assess survival of bacteria [25, 26]. The lengthy process of CFU determination did not provide opportunity to examine effector T cell activity in detail. However, the novel immune cell overlay model designed to examine the effector T cell response to BCG infected MoMΦ, provided a method to examine the effector T cell recall response elicited from APC pulsed with Ags. To overcome the time required to determine viability by the CFU method, we adapted the method by Kralik et al. [17] as an expedient alternative way to distinguish live from dead *Map*. This approach, which combined a membrane impermeant fluorescent dye (PMA) with quantitative PCR, eliminated the need to determine CFU counts for quantifying *Map* in samples obtained from food and the environment [17, 27, 28]. Initial studies were conducted to determine whether this PMA method could be adapted for use in the present study. As illustrated in Figure 3, we verified it is possible to use qPCR with a single copy of the *Map* specific gene, F57, as a probe to generate a curve with a large dynamic range (4 × 10^7^ copies of F57 to 4 copies). We then conducted studies to determine if there was sufficient resolution to distinguish differences in the relative percent of live bacteria in preparations of *Map* containing known proportions of live and dead bacteria, starting with DNA isolated from pure cultures of *Map* (Figure 3A). There was a background showing the PMA didn’t block all the DNA from dead bacteria. However, the mixtures of live and dead bacteria fit on the curve, with sufficient difference in C_T_ values between dead and live bacteria to distinguish and enumerate differences in the percent of live bacteria present in a mixture of dead and live bacteria. *Map* DNA isolated from infected MoMΦ also fit on the standard curve, but shifted values toward fewer copies of F57 and higher C_T_ values (Figure 3A).

We next determined if a sufficient number of bacteria were taken up by MoMΦ in a 3 h time frame to be quantitated using the PMA method. A two well culture slide was prepared with MoMΦ and infected with *Map* K10_GFP_ under the same culture conditions used to set up infected MoMΦ for the viability assay (see "Materials and methods"). Confocal microscopy showed efficient bacterial uptake following 3 h of incubation (Figure 3B), even without a centrifugation step to concentrate the bacteria to the cell surface of MoMΦ for uptake.

The qPCR assay showed the number of bacteria taken up was sufficient to consistently determine the relative percent of live *Map* present within MoMΦ after 3 h of incubation (Figure 3D). Importantly, the study showed there was minimal killing of bacteria mediated by MoMΦ during the 6 h incubation period with MoMΦ cultured alone or in the presence of mdPBMC from control steers. (Figure 3D). We concluded that the dynamic range between live and dead bacteria isolated from MoMΦ was adequate for studying the intracellular killing activity of effector CD4 and CD8 T cells proliferating in response to stimulation with MoDC, pulsed with Δ*Map*/*relA* or MMP.

### Analysis of the proliferative response and functional activity of mdPBMC from the vaccinated and control steers stimulated by MoDC pulsed with Δ*Map*/*relA* or MMP

As reported previously, extensive studies with PBMC from the vaccinated steers showed a CD4 CD8 T cell recall response could be elicited ex vivo by stimulation with Δ*Map*/*relA* [11, 12]. The studies demonstrated an equivalent recall response could be elicited with mdPBMC using bDC or MoDC pulsed with Δ*Map*/*relA* or MMP. The response was MHC restricted [12]. As reported here, studies were extended to determine if the functional activity of the proliferating cells could be determined using a modification of the intracellular killing assay described by Worku and Hoft [25] and a PMA-based method to distinguish live from dead *Map* present in *Map*-infected MoMΦ [17].

**Figure 4:**
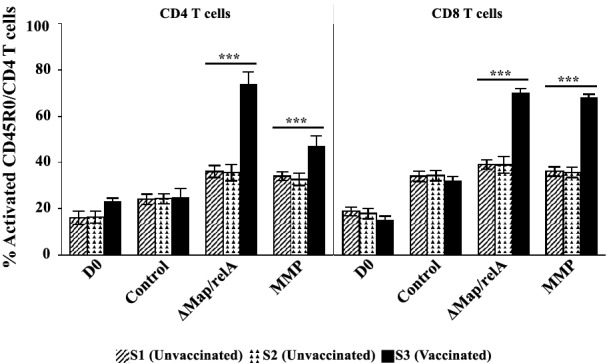
**Two-way ANOVA comparison CD4, CD8 T cell response unvaccinated and vaccinated steers to Ag-pulsed MoDC.** The factors were steer-status (three levels: S1-unvaccinated, S2-vaccinated, S3-vaccinated) and treatments (four levels: D0, Control, ∆*Map*/*relA*, MMP). Tukey HSD (overall α = 0.05) was applied post-hoc to determine the statistical significance of comparisons of interest. For each T cell type (CD4 and CD8), the mean and standard deviation for each treatment effect (*n* = 6 independent experiments) within each steer-status is shown. The two-way ANOVAs for CD4 and CD8 T cells were significant (*F*_*CD4*_ = 158.2463, *F*_*CD8*_ = 379.0110; each, *P* < 0.0001) and each included a significant interaction effect between treatments and steer-status (*F*_*CD4*_ = 56.3527, *F*_*CD8*_ = ;165.4418; each, *P* < 0.0001). For both CD4 and CD8 T cells, the mean effects of ∆*Map*/*relA* and MMP in vaccinated steer S3 were greater than in unvaccinated steers S1 and S2 (each, *P* < 0.0001); significant differences not detected between unvaccinated steers in the mean effects of *∆Map*/*relA* (*P*_*CD4*_ = 1.0000, *P*_*CD8*_ = 1.0000) or MMP (*P*_*CD4*_ = 0.9997, *P*_*CD8*_ = 1.0000).

We first examined the effector T cell recall response to MoDC pulsed with Δ*Map*/*relA* and MMP (Figure 4). The proliferative responses to Δ*Map*/*relA* and MMP were very similar. We used *Map* infected-MoMΦ as targets for the study. The preliminary studies revealed effector T cells from the vaccinated steer were able to kill intracellular bacteria whereas there was little killing activity observed with cells from the control steers (data not shown). Further testing with MoDC pulsed with MMP yielded similar results (data not shown). To complete and validate the observations, the entire protocol (outlined in the protocol, Figure 1) was repeated 6 times in parallel with MoDC pulsed with Δ*Map*/*relA* in one set and a second set pulsed with MMP (Figure 3D). As observed in the preliminary studies to set up the assay, there was limited intracellular killing by effector cells from the control steer mdPBMCs stimulated once with MoDC pulsed with Δ*Map*/*relA* or MMP (Figures 3C and D). The relative concentration of live bacteria detected in infected MoMΦ incubated with unstimulated mdPBMC from the control steers was similar to the concentration present in infected MoMΦ incubated alone (Figure 3A). In contrast, few viable *Map* were present in infected MoMΦ incubated with Ag-primed mdPBMC from the vaccinated steer. The repeat of the assay 6 times for both Δ*Map*/*relA* and MMP, demonstrated the consistency in results from test to test as summarized in Figure 3D (*P* < 0.0001).

### Analysis of the proliferative and functional activity of naïve mdPBMC from unvaccinated steers stimulated with bDC and MoDC pulsed with Δ*Map*/*relA* and MMP

**Figure 5:**
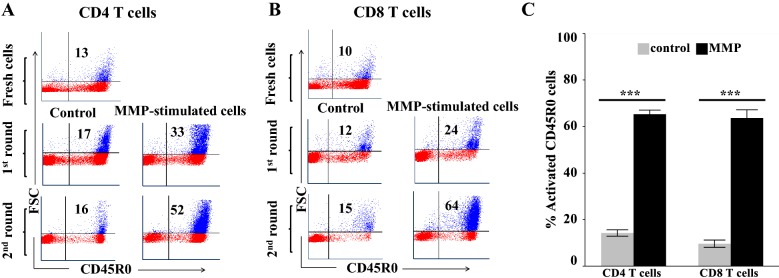
**Comparison response CD4, CD8 T cells from unvaccinated steers stimulated with MMP-pulsed bDC and MoDC.** **A** Representative flow cytometric profiles showing level of CD4 T cell proliferation following one and two rounds of antigenic stimulation by Ag-pulsed DC. **B** Representative flow cytometric profiles showing level of CD8 T cell proliferation following one and two rounds of antigenic stimulation by Ag-pulsed DC. **C** Two-way ANOVA with interaction. The factors were type of T cell (two levels: CD4, CD8) and MMP-stimulation (two levels: control, MMP). Tukey HSD (overall α = 0.05) was applied post-hoc to determine the statistical significance of comparisons of interest. The mean and standard deviation for each treatment effect for each type of T cell is shown (*n* = 4 unvaccinated steers). The two-way ANOVA was significant (*F* = 732.6776; *P* < 0.0001). The mean effect of MMP stimulation was significant (*F*_*stimulation*_ = 2188.613, *P* < 0.0001) but not dependent on T cell type (*F*_*interaction*_ = 1.33; *P* = 0.2092). The mean effect of T cell type was small but significant (CD4 effect estimate = 1.55 ± 0.56; *F*_*CD*_ = 7.6591, *P* = 0.0170).

**Figure 6:**
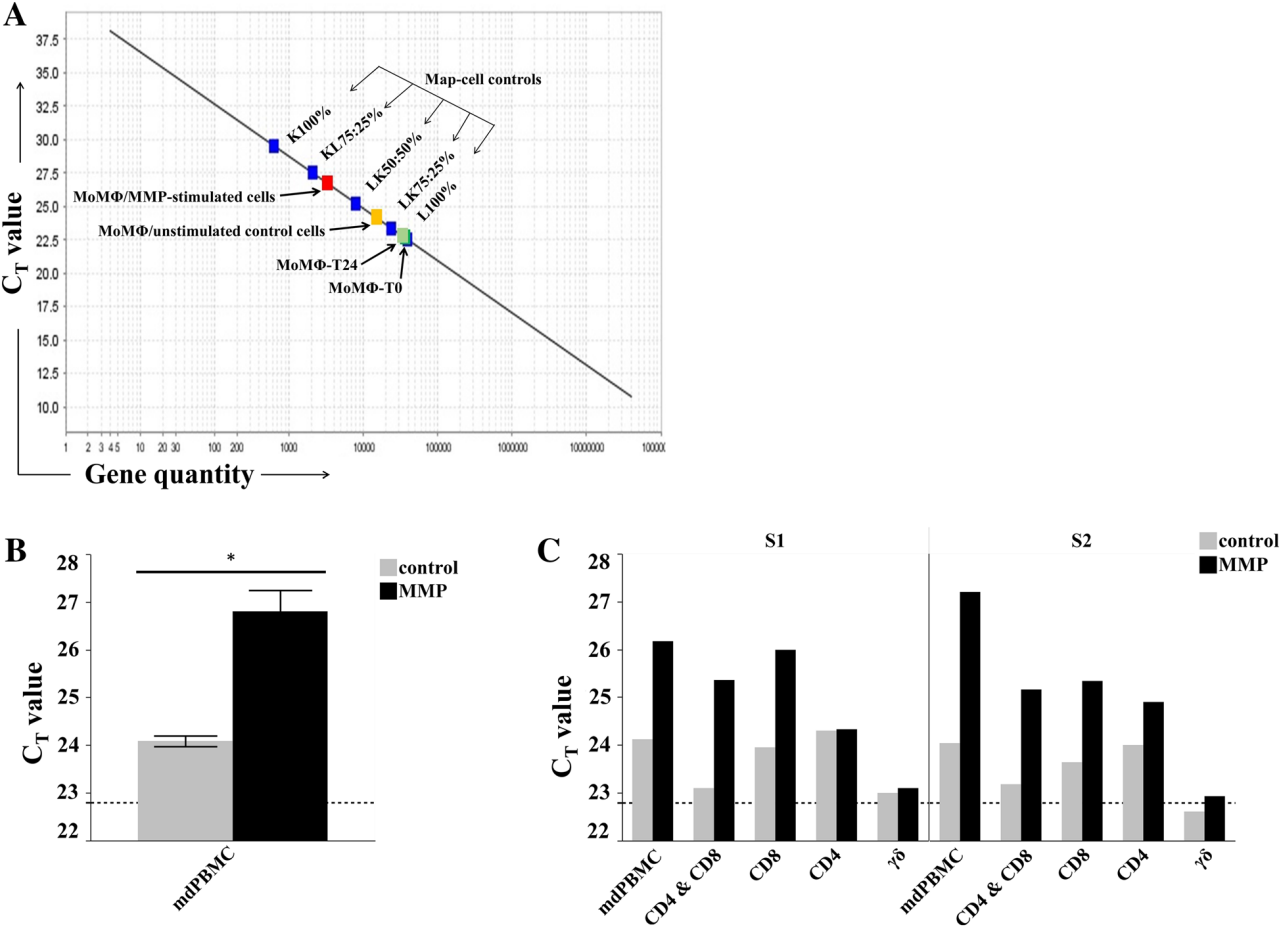
**Comparison of the killing activity of CD4, CD8, and γδ T cells. A** Representative set of results illustrated with the C_T_ values obtained with unseparated cell populations. The C_T_ values represent the average of duplicate preparations of DNA run at the same time. **B** A two-way ANOVA for the effect of mdPBMC stimulation (two levels: control, MMP), reponses grouped by unvaccinated steers (*n* = 4). The mean and standard deviation for each stimulation level (control, MMP) of mdPBMC from four unvaccinated steers. The effect of MMP stimulation was significant (*F*_*stimulation*_ = 130.4208; *P* = 0.0014). **C** No statistics were applied since only two of the four unvaccinated steers were used to obtain data on separated cell subsets.

The question of why the Δ*Map*/*relA* mutant could not establish a persistent infection was answered [10]. Vaccination elicited a CTL response that cleared the infection with Δ*Map*/*relA*. The ex vivo findings indicated that the effector activity of CD4 and CD8 T cells from the vaccinated steer, proliferating in response to stimulation with APC pulsed *Map*/*relA* and MMP, was directed towards MMP (Figures 3 and 4). To extend these findings, we conducted follow up studies entirely ex vivo. If successful, we postulated this would afford a way to study the activity of APC at the time of Ag presentation and also responses of CD4 and CD8 T cells, differentiating in response to signaling by APC pulsed with Ags. As mentioned in the methods, we modified the stimulation protocol to include two rounds of stimulation of mdPBMC from the four unvaccinated steers, starting with stimulating mdPBMC with bDC pulsed with Δ*Map*/*relA* or MMP (first round of stimulation) and 6 days later stimulation with MoDC pulsed with Δ*Map*/*relA* or MMP (second round of stimulation). Comparison of the proliferative response to Δ*Map*/*relA* and MMP showed the responses were equivalent (data not shown). Based on these observations, further studies were conducted just with MMP. We repeated the complete protocol with the four steers. As shown in Figures 5A and B, a proliferative CD4 and CD8 T cell response to MMP was evident by 6 days. However, cell proliferation was not sufficient for analysis of CTL activity. There were not enough activated cells present after one round of stimulation to accurately perform the killing assay. After a second round of stimulation, there was robust proliferation of both CD4 and CD8 T cells, providing enough cells to perform the CTL assay (Figures 5C, 6A and B).

### The essential need for CD4 and CD8 T cell cognate recognition of MMP peptides presented by APC for development of CD8 CTL against *Map*

All the previous studies up to this time were conducted with mdPBMC because it was unknown whether the response to Ag peptides, processed and presented by APC pulsed with Δ*Map*/*relA* or MMP, required cognate recognition of Ag peptides by CD4 and CD8 T cells to generate CTL activity. Also, it was assumed but not verified that CTL activity was primarily associated with CD8 T cells. To address these unknowns, a series of experiments were conducted with freshly isolated mdPBMC from the 2 steers. The first experiments focused on determining whether both CD4 and CD8 T cells needed to be present at the time of Ag presentation to generate CTL activity. The second set of experiments were focused on demonstrating whether CTL activity was primarily associated with CD8 T cells proliferating in response to Ag presented by APC. mdPBMC from 2 of the unvaccinated steers were depleted of either CD4 or CD8 T cells and then subjected to 2 rounds of stimulation with APC pulsed with MMP. Preparations of unseparated mdPBMC, unstimulated and stimulated, were used as controls. There was limited cell proliferation in cultures of mdPBMC depleted of CD4 or CD8 T cell following two rounds of stimulation with APC pulsed with MMP (data not shown). In contrast, there was robust proliferation of CD4 and CD8 T cells in the unseparated preparations of mdPBMC following two rounds of stimulation (results similar to those shown in Figure 5C). Following demonstration that both CD4 and CD8 T cells must be present for generation of a proliferative and CTL response to MMP, we used the two steers to determine which cell subset(s) possessed CTL activity. Negative and positive selection were used to obtain preparations of Ag-primed mdPBMC containing CD4 and CD8 T cells, CD4 T cells, CD8 T cells alone or only γδ T cells. Preparations of unseparated unstimulated and stimulated mdPBMC were used as controls. The proliferation (not shown) and killing activity of unseparated preparations mdPBMC stimulated with APC, pulsed with MMP, were similar to the results obtained with all 4 steers (compare Figure 6C with Figure 6B). Killing activity was less but clearly evident in cell preparations containing both CD4 and CD8 T cells and CD8 T cells alone (Figure 6C). Little or very limited killing activity was evident in cell preparations containing CD4 T cells. No killing activity was evident in the cell preparations containing γδ T cells (Figure 6C).

### Role of the perforin/ GnzB pathway in intracellular killing of *Map*

**Figure 7:**
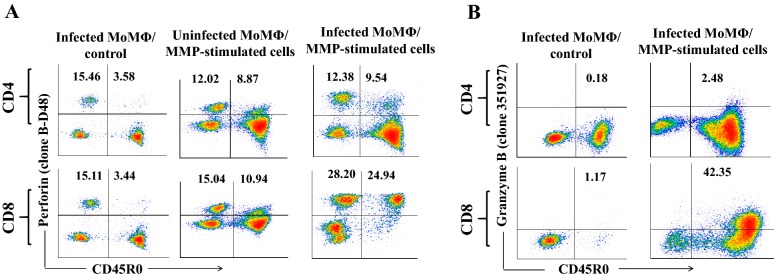
**Flow cytometric analysis showing expression of newly synthesized perforin, GnzB in CD4, CD8 T cells. A** Newly synthesized perforin was highly expressed in CD8 T cells from unvaccinated steers with low expression in CD4 T cells (right set of graphs). Newly synthesized perforin was below the limits of detection in primed mdPBMC incubated with uninfected MoMΦ (center set of graphs). **B** Expression of GnzB in infected MoMΦ was low in CD4 T cells. GnzB was present in approximately half of the CD8 T cells.

Extensive studies have shown intracellular killing of intracellular bacteria and protozoan parasites is mediated by perforin, Gnz B, and granulysin [29, 30]. We were interested in determining whether perforin and granzyme B were also involved in the intracellular killing of *Map*. A mAb reactive with bovine granulysin was not available. Nonetheless, we verified a mAb to human perforin (δG9) identified a conserved epitope on bovine perforin (data not shown) [31]. Screening of other commercially available mAbs for cross reactivity with bovine perforin revealed B-D48, a mAb that recognizes a different conserved epitope expressed on the native and newly synthesized forms of perforin, reacted with bovine perforin [32, 33]. This mAb was selected for further analysis to determine the pattern of expression of newly synthesized perforin in CD4 and CD8 T cells at the end of the killing assay. In addition, a recent report showed a mAb to human GrzB, 351927, cross reacted with bovine GrzB [34]. We verified the mAbs cross-reactivity with bovine GrzB and then used it in the present study. The primed CTL were incubated with uninfected and infected MoMΦ to determine if recognition of infected cells is necessary to stimulate synthesis of new perforin (Figure 7A). Newly synthesized perforin was only detected in memory CD4 and CD8 T cells stimulated with MMP-pulsed MoDC, with the highest level of expression was in CD8 T cells (Figure 7A). Comparison of expression of GnzB showed expression was low in activated memory CD4 T cells and high in half the activated memory CD8 T cells (Figure 7B).

### Infected cells do not undergo apoptosis during intracellular killing of *Map*

**Figure 8:**
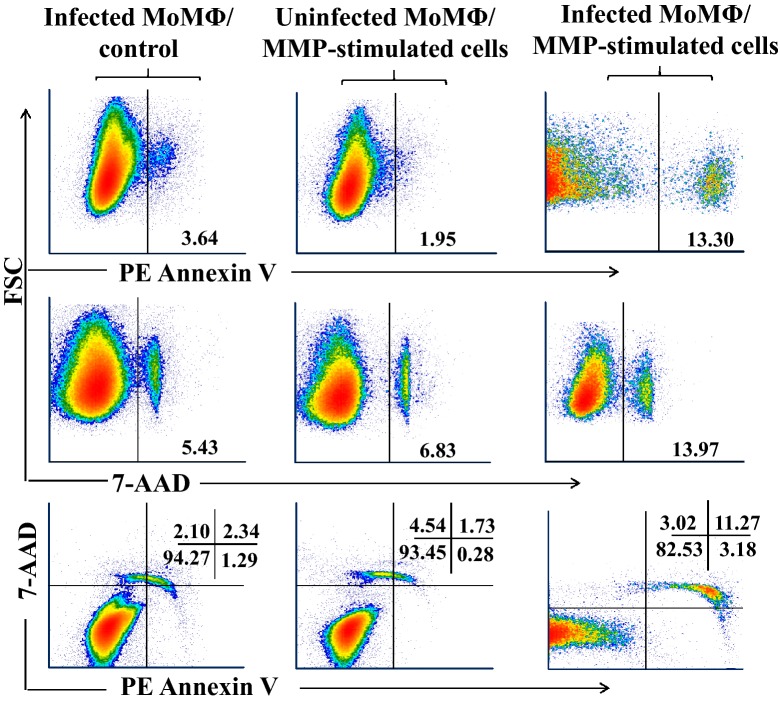
**Comparison of apoptosis in uninfected and infected MoMΦ incubated with MMP-stimulated mdPBMC.** The frequency of apoptosis was low in infected MoMΦ incubated with naïve unprimed mdPBMC and in uninfected MoMΦ incubated with MMP-stimulated mdPBMC. Although a small increase in apoptosis was observed in infected MoMΦ incubated with MMP-stimulated mdPBMC, the majority of cells in each cell preparation were neither necrotic nor apoptotic.

Studies of the events associated with killing target cells by CTL have shown that introduction of GrzB into the cytoplasm of tumor cells or target cells infected with virus activates caspases that initiate apoptosis and cell death [35, 36]. The role of apoptosis in the intracellular killing of bacteria, however, has remained unresolved. Previous studies of intracellular killing of *Mtb* by Thoma-Uzynski et al. [37] showed “host cell apoptosis is neither sufficient nor necessary for CD8^+^ CTL to kill intracellular *Mtb*” [37]. We used a FC assay to determine if apoptosis is associated with intracellular killing of *Map*. Comparison of the frequency of necrotic apoptotic cells in preparations of infected MoMΦ incubated alone vs necrotic apoptotic cells in preparations of uninfected MoMΦ incubated with primed mdPBMC showed the frequencies were similar and low. The frequency was a little higher in preparations of infected MoMΦ incubated with primed mdPBMC (Figure 8). However, the majority of infected MoMΦ, incubated under the different conditions, were not necrotic or apoptotic even after 24 h of incubation. Most of the cells had intact membranes as detected with 7-AAD. An additional experiment with infected MoMΦ incubated for 3 days with primed mdPBMC showed the majority of infected MoMΦ were still alive after the intracellular killing event.

## Discussion

The studies presented here were focused on analysis of the immune response to *Map* using the natural host. The initial studies leading up to the present studies showed all animals exposed to *Map* under experimental conditions at birth, develop an infection that elicits an immune response that controls but doesn’t eliminate the pathogen [38, 39]. Flow cytometric analysis of the immune response to *Map* and soluble *Map* Ags ex vivo demonstrated an immune response to *Map* could be detected 3–5 months post exposure that included CD4 and CD8 T cells [39]. γδ T cells did not exhibit an Ag specific response. Further analysis with a cannulated ileum model demonstrated that *Map* introduced directly into the ileum are rapidly taken up and disseminated to other sites without establishing detectable lesions in the ileum containing *Map*, as detected by biopsy. In fact, no lesions were detectable up to 18 months post exposure [24]. A follow up study comparing the immune response to human and bovine isolates of *Map* showed no difference in the immune response to *Map*. The one interesting comparative observation was that it appeared the CD8 T cell response in experimentally infected calves was delayed, reaching a maximum at 12 months [40]. Development of a method to use site directed mutagenesis provided opportunity to look at the immune response to *Map* in greater detail and determine if any gene product was essential for establishing a persistent infection. Three genes were selected for analysis: *relA*, demonstrated to impair survival of *Mtb* in a mouse model [41]; *pknG*, shown to affect survival of BCG in macrophages [42]; and *lsr2*, shown to be involved in mediating multi-drug resistance to antibiotics [43]. In vivo studies revealed, similar to the studies in mice with *Mtb*, that deletion of *relA* impaired survival [9, 10]. Comparison of survival with *Map* at necropsy showed wild type *Map* was readily detected in multiple tissues whereas Δ*Map*/*relA* was not detected in any tissue using culture and PCR. These findings and the ex vivo studies indicated there was a clear difference in the immune response to the Δ*Map*/*relA* mutant and suggested that deletion of the gene allowed for the development of an effector T cell response that cleared the infection. To investigate the immunological mechanisms accounting for clearance of the mutant, two steers were vaccinated with Δ*Map*/*relA* and maintained as a source of cells to characterize the effector T cells ex vivo. We hypothesized the difference in the immune response to *Map* and Δ*Map*/*relA* might involve signaling mediated through antigen presenting cells (APC). To examine this possibility we developed and used a mAb to CD209 (uniquely expressed on bDC, MoDC, and MoMΦ) to phenotypically identify and compare the capacity of bDC, MoDC, and MoMΦ to process and present Ags to effector memory T cells, elicited by vaccination with Δ*Map*/*relA* [12]. The studies revealed the complexity of the immune response to the mutant. The study revealed bDC, MoDC, and MoMΦ were essentially equal in their capacity to process and present Ag to naïve T cells. Of special interest, all three APCs presented antigenic peptides in context of MHC I and II molecules simultaneously and provided signaling that elicited a CD4 and CD8 proliferative response.

Ancillary studies conducted to determine which *Map* proteins might be involved in the immune response to *Map*, included examining the proliferative response to the whole and truncated forms of the 35 kDa membrane protein, MMP [13, 44]. A proliferative response was obtained with the whole and truncated forms of the protein (unpublished). As reported here, further studies conducted to determine if MMP was a component of the recall response, elicited with Δ*Map*/*relA*, demonstrated it was included as a component of the immune response. Comparative studies demonstrated equivalent recall responses were obtained with Δ*Map*/*relA* and MMP with all three APCs [12]. The recall response was MHC restricted [12].

In the present study, we initially used the vaccinated and control unvaccinated steers to examine the functional activity of memory T cells proliferating in response to Ag presentation by MoDC pulsed with Δ*Map*/*relA* and MMP. To conduct these studies, a viability assay was developed based on studies by Worku and Hoft, using patients with tuberculosis and control subjects vaccinated with the BCG [25]. In addition, we adapted an expedient method for distinguishing live from dead bacteria to obviate the problem of using CFU as a read out of bacterial viability [17]. Although the conditions for blocking all gene sites was not optimal for PMA, it was sufficient to be able to detect differences in live vs dead bacteria. The scale showed where the residual DNA from dead cells falls on the curve. The wide dynamic range between live and dead cells was consistent for distinguishing between DNA from live and dead bacteria. Comparison of the recall and functional activity of memory CD4 and CD8 T cells proliferating in response to Ag presentation by MoDC pulsed with Δ*Map*/*relA* and MMP revealed equivalent CTL activity was elicited with Δ*Map*/*relA* and MMP. The results proved highly reproducible using APC pulsed with either Δ*Map*/*relA* or MMP. A singular observation made throughout the studies was, regardless of the route of exposure to *Map*, Δ*Map*/*reA*, or MMP, exposure resulted in development of a CD4 CD8 T cell response [9, 12, 39, 40]. This raised two questions. Is cognate recognition of Ags by CD4 and CD8 a requirement for development of CTL activity? And, do both CD4 and CD8 T cells develop CTL activity? Only one of these questions could be answered with further studies using vaccinated animals. This prompted us to explore the possibility of developing an assay to study the full CTL response ex vivo. The results showed equivalent Ag presentation could be achieved with bDC and MoDC pulsed with either Δ*Map*/*reA*, or MMP. This suggested APC could be used to develop a method to study the full immune response to Ags ex vivo. If successful, it would be possible to study mechanisms of Ag processing and presentation by APC to a relevant pathogen. It would also make it possible to study the importance of cognate recognition of antigenic peptides presented in context of MHC class I and II molecules in development of CTL activity.

As reported here, four unvaccinated steers were used to modify the protocol for studying the recall response starting with stimulation of naïve T cells with bDC pulsed with Δ*Map*/*relA* or MMP followed by a second round of stimulation with MoDC pulsed with Δ*Map*/*relA* or MMP. After demonstrating equivalent responses occurred with Δ*Map*/*relA* and MMP, further studies were focused on analysis of the immune response to MMP. The first question of interest was whether the CD4 and CD8 T cells needed to be present in a culture to generate CTL. The studies conducted to this point in the investigation involved use of mdPBMC. Little or no response was observed if CD4 and CD8 T cells were separated from preparations of mdPBMC before primary Ag presentation by bDC followed by secondary stimulation with MoDC. In contrast, a full CTL response was consistently obtained if unseparated mdPBMC were used. The second question of interest was which T cell subset possessed the CTL activity. The unproven supposition was that CTL activity was primarily attributable to CD8 T cells. To answer this question, mdPBMC primed with APC pulsed with MMP, were separated into 4 populations before incubating with infected MoMΦ target cells. Cell preparations containing CD4, CD8, and CD4/CD8 were obtained by negative selection. The cell preparation containing CD8^+^ and CD8^−^ γδ T cells was obtained by positive selection. Comparison of CTL activity showed unseparated mdPBMC contained the highest level of CTL activity followed by the cell preparations containing both CD4 and CD8 T cells and CD8 T cells. Minimal or no activity was observed with separated CD4 T cells or γδ T cells. NK cells were not evaluated for potential CTL activity in this study. Repeat studies showed they only comprised ~5% of cells present in mdPBMC stimulated with Ag-pulsed bDC and MoDC. The results demonstrated the importance of the interaction of CD4 and CD8 T cells in the generation of CTL. Further studies are needed to detail the signaling associated with interaction of CD4 and CD8 T cells during Ag presentation.

Extensive studies conducted to elucidate the mechanisms used by CTL to kill intracellular bacteria and protozoan parasites have shown perforin, GrzB, and granulysin play a role in killing [30, 45]. Our studies clearly show perforin and GrzB are involved in killing of *Map*. Further studies should show granulysin is also involved. An immune synapse develops on contact of CTL with the infected cells. Cytotoxic granules migrate to the synapse allowing perforin molecules to intercalate into the membrane of the target cell and form pores through which granzymes and granulysin are introduced into the cytoplasm. Granulysin disrupts the integrity of the bacterial cell membrane allowing entrance of GrzB and death of the bacteria [30]. Analysis of effector cells after incubation with uninfected and infected MoMΦ showed CTL contact with infected MoMΦ is essential for killing. Newly synthesized perforin was present in CD8 CTL incubated with infected MoMΦ. Only a few CD4 T cells contained newly synthesized perforin. In contrast, there was minimal or no increase in newly synthesized perforin in primed CD8 CTL incubated with uninfected MoMΦ. GrzB was detectable in half of the CD8 CTL with no detectable level in CD4 T cells. These results indicate the intracellular killing of *Map* is associated with upregulation of both perforin and GrzB, mainly in CD8 CTL.

It has remained unclear as to whether apoptosis of the target cells, mediated by the perforin–granzyme pathway, is an essential component of the mechanisms of intracellular killing of bacteria. Studies by Thoma-Uszynski et al. [37] have suggested killing of *Mtb* is independent of apoptotic death of infected target cells. We conducted studies to examine this issue. Analysis of apoptosis showed the majority of infected MoMΦ were still viable after incubation with primed mdPBMC. The average percentages of apoptotic cells observed in infected MoMΦ co-cultured with Ag-primed mdPBMC was 13% compared to ~4% in infected MoMΦ co-cultured with control mdPBMC and ~2% in uninfected MoMΦ co-cultured with Ag-primed mdPBMC. This is in contrast to CTL mediated killing of virus infected and cancer cells where apoptosis is clearly involved [46]. Introduction of GnzB leads to immediate initiation of apoptosis and death of the target cell. The majority of target cells infected with *Map* were still alive even after 3 days of incubation with Ag-primed mdPBMC. Further studies are needed to explain this apparent paradox. The interaction between *Map* and macrophages is a complex process affected by the lethal signals released by the CTL involved in the bacterial killing and cell apoptosis. Further studies of the roles of apoptosis in defense against *Map* infection are needed to fully explain the results obtained by Thoma-Uszynski et al. [37] and the results obtained in the present study.

In summary, we have used the natural host to gain a better understanding of the immune response to *Map* and candidate vaccines. Data obtained thus far have shown *relA* plays a central role in the ability of *Map* to establish a persistent infection and that deletion of *relA* leads to immune elimination of the mutant. Ex vivo analysis has shown MMP is a component of the immune response to Δ*Map*/*relA*. An equivalent CD8 CTL response is elicited with APC pulsed with Δ*Map*/*relA* or MMP. Simultaneous presentation of antigenic peptides to CD4 and CD8 by Ag pulsed bDC and MoDC is essential for generation of CTL. CTL activity is mediated through the perforin/granzyme pathway. In contrast to CTL activity against virus infected cells and cancer cells, intracellular killing of bacteria occurs with limited induction of apoptosis. The assays developed to conduct the studies ex vivo provide opportunities to study the potential efficacy of candidate vaccines as well as opportunities to study Ag presentation by DC and consequent proliferation and differentiation of CD4 and CD8 T cells with different functional activity.
